# Effects of climatological parameters in modeling and forecasting seasonal influenza transmission in Abidjan, Cote d’Ivoire

**DOI:** 10.1186/s12889-016-3503-1

**Published:** 2016-09-13

**Authors:** A.K. N’gattia, D. Coulibaly, N. Talla Nzussouo, H.A. Kadjo, D. Chérif, Y. Traoré, B.K. Kouakou, P.D. Kouassi, K.D. Ekra, N.S. Dagnan, T. Williams, I. Tiembré

**Affiliations:** 1 Department of Epidemiology, Institut National d’Hygiène Publique, BP V 14, Abidjan, Côte d’Ivoire; 2 Training and Research Unit of Medical Sciences, Department of Public Health and Community Medicine, Félix Houphouët-Boigny University, BP V 34, Abidjan, Côte d’Ivoire; 3 Influenza Division, U.S. Centers for Disease Control and Prevention, 1600 Clifton Road, Atlanta, GA 30329-4027 USA; 4 Department of Virology, Respiratory Diseases, Pasteur Institute, 01 BP 490, Abidjan 01, Côte d’Ivoire; 5 Department of Public Health and Community Medicine, Alassane Ouattara University, BP V 18, Bouaké, Côte d’Ivoire

**Keywords:** Modeling, Influenza, Climatological parameters, Abidjan

## Abstract

**Background:**

In temperate regions, influenza epidemics occur in the winter and correlate with certain climatological parameters. In African tropical regions, the effects of climatological parameters on influenza epidemics are not well defined. This study aims to identify and model the effects of climatological parameters on seasonal influenza activity in Abidjan, Cote d’Ivoire.

**Methods:**

We studied the effects of weekly rainfall, humidity, and temperature on laboratory-confirmed influenza cases in Abidjan from 2007 to 2010. We used the Box-Jenkins method with the autoregressive integrated moving average (ARIMA) process to create models using data from 2007–2010 and to assess the predictive value of best model on data from 2011 to 2012.

**Results:**

The weekly number of influenza cases showed significant cross-correlation with certain prior weeks for both rainfall, and relative humidity. The best fitting multivariate model (ARIMAX (2,0,0) _RF) included the number of influenza cases during 1-week and 2-weeks prior, and the rainfall during the current week and 5-weeks prior. The performance of this model showed an increase of >3 % for Akaike Information Criterion (AIC) and 2.5 % for Bayesian Information Criterion (BIC) compared to the reference univariate ARIMA (2,0,0). The prediction of the weekly number of influenza cases during 2011–2012 with the best fitting multivariate model (ARIMAX (2,0,0) _RF), showed that the observed values were within the 95 % confidence interval of the predicted values during 97 of 104 weeks.

**Conclusion:**

Including rainfall increases the performances of fitted and predicted models. The timing of influenza in Abidjan can be partially explained by rainfall influence, in a setting with little change in temperature throughout the year. These findings can help clinicians to anticipate influenza cases during the rainy season by implementing preventive measures.

**Electronic supplementary material:**

The online version of this article (doi:10.1186/s12889-016-3503-1) contains supplementary material, which is available to authorized users.

## Background

The seasonality of influenza has been well described in temperate regions where influenza activity typically coincides with winter [1–4]. In these temperate countries, low temperatures, low relative or absolute humidity, minimal solar radiation are associated with influenza related deaths [3, 5, 6]. In subtropical regions, studies demonstrated a strong correlation between relative humidity, ambient temperature and influenza viruses’ circulation and their considerable impact on the occurrence of influenza epidemics [7, 8]. In temperate and subtropical countries, the effects of these climatological parameters on the transmission of influenza seem to be elucidated, but it remains unclear in tropical countries, especially in Africa. Indeed, the seasonality of influenza is difficult to predict [1, 9] and the circulation of influenza viruses has an irregular pattern with no discernable peaks throughout the year in the tropical countries [1, 2, 4, 10]. Furthermore, some authors demonstrated that the transmission dynamics of influenza and other seasonal respiratory infections coincides with rainfall and higher relative humidity [1, 2, 9–12]. Lowen highlighted a high prevalence of influenza transmission by direct contact in tropical regions [6]. But, Alonso showed that temperature and humidity are more favorable to influenza transmission in tropical regions compared to transmission by direct contact [13]. Moreover, the influenza viruses are able to infect host and spread, if climatological and environmental parameters promote their survival, transmission and seasonality [14]. The objective of this study is to determine is there is any correlation between climatological parameters and influenza infection and to what extent these parameters are useful to forecast influenza transmission in Abidjan, Cote d’Ivoire.

## Methods

### Study design

A retrospective time series study was conducted in Abidjan to determine any correlation between climatological parameters and the weekly incidence of influenza cases.

### Climatological data

Weekly cumulative rainfall, average weekly relative humidity, and average weekly ambient temperature were obtained from the database of the Airport Operating Development Aviation and Meteorology Company (SODEXAM). These data were collected by this company at its weather stations in Abidjan from January 2007 to December 2012. Data from 2007 to 2010 were used for model fitting and those from 2011 to 2012 for forecasting.

### Epidemiological and Virologic data

We conducted a review of the database of the influenza surveillance network of the National Institute of Public Hygiene (INHP) from 2007 to 2012. So, we collected the weekly positive cases of influenza from all the 15 sentinel sites of Abidjan. Following WHO guidelines and case definitions, the surveillance staffs at sentinel sites obtained specimens for influenza testing. Each sentinel site enrolled, the first three patients that presented with signs of influenza-like illness during the week. Also for Severe Acute Respiratory Infections (SARI), all the cases screened by physicians each week and that met the case definitions for SARI were enrolled and sampled. The samples are packed in coolers containing ice packs and shipped to the laboratory within 48 h of sampling. The National Influenza Centre (NIC) laboratory located at the Pasteur Institute of Côte d’Ivoire tested the influenza specimens. The method used for the confirmation of influenza positive cases at the NIC combined conventional RT-PCR, real-time RT-PCR and virus isolation on MDCK cell culture [11].

### Statistical analysis

In order to model the weekly incidence of influenza transmission, we used autoregressive integrated moving average models (ARIMA). We firstly depicted the curves of weekly incidence of influenza cases, rainfall, relative humidity and ambient temperature. Then, we made a breakpoint variable that was introduced into the models for the correction of the process that occurred at epidemiological week 19 of 2009 following the sudden increase of positive influenza strains that were isolated as a result of the occurrence of the 2009 Influenza A (H1N1) pandemic, the installation of new sentinel sites, and the acquisition of new diagnosis techniques for the NIC (real-time RT-PCR).

We developed univariate ARIMA models of the weekly incidence of influenza cases that depended only on its past values, residuals and the breakpoint variable. To assess the association between the weekly incidence of influenza cases and rainfall, relative humidity and ambient temperature, at different lags (prior epidemiological weeks), we used the cross-correlation test of Pearson. ARIMAX multivariate models were developed by introducing climatological parameters correlated at different lags. Climatological variables that were not significantly correlated with the main series at different lags were not included in the multivariate models. The correlated climatological variables were included one by one, before combining them together in the multivariate models. We used as dependent variable the weekly incidence of influenza cases and as independent variables the weekly cumulated rainfalls, average weekly relative humidity and average weekly ambient temperature (Additional file 1).

We used 208 observations (one for each week during January 1st, 2007 to December 31st, 2010: 2007EW1 to 2010EW52) to fit the ARIMA models and 104 observations (2011EW1 to 2012EW52) for forecasting (1-week-ahead forecast) the weekly incidence of influenza cases (Additional file 2). Thus, the best multivariate reference model was used to explain and predict influenza transmission in Abidjan. For the fitting and forecasting of models, we applied the brief description of the methods of Box-Jenkins (1976) for ARIMA modeling. Model was selected as best performing based on having the lowest values for Akaike (AIC) and Schwarz (BIC) Information Criterion and significant Portmanteau test for residuals’ autocorrelation. For all statistical tests, *p*-values < .05 were considered to be statistically significant. Data were analyzed using statistical software *Stata MP 12.0*, *StataCorp LP*, *College Station*, *Texas*.

## Results

### Description

**Fig. 1:**
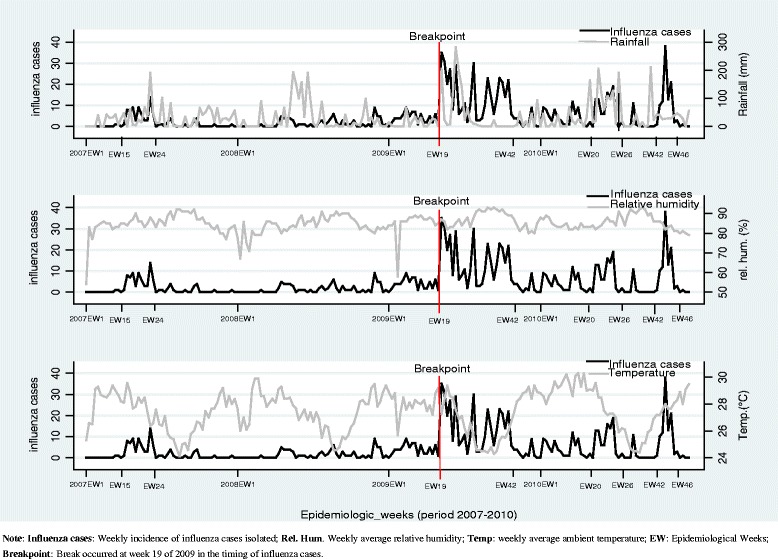
Weekly incidence of influenza cases and weekly rainfall, weekly average relative humidity, and weekly average ambient temperature observed in Abidjan, Côte d’Ivoire from 2007 to 2010

From January 2007 to December 2010, the sentinel surveillance network in Abidjan reported 921 influenza positives cases. The weekly incidence of influenza cases showed no clear seasonality but was marked by two phases. The first phase showed few cases of influenza from 2007EW1 to epidemiological week 19 of 2009, i.e. the start of the 2009 H1N1 pandemic. The second portion was more extensive until 2010EW52. This influenza activity was higher in 2007 from EW15 to EW24, in 2009 from EW19 to EW42 and in 2010, from EW20 to EW26 and from EW42 to EW46 (Fig. 1).

Unlike influenza activity, the seasonality of the rainfalls, relative humidity and ambient temperature were well defined. Also, the high influenza activity was often accompanied by an increase in rainfall and relative humidity and a fall in ambient temperature. However in the last period (2010EW42 to 2010EW46), the high influenza activity coincided with an increase of ambient temperature and decrease of rainfall and relative humidity (Fig. 1).

### Bivariate and multivariate analysis

Significant cross-correlation was observed between the weekly incidence of influenza cases and weekly cumulated rainfalls at lags (current and prior weeks) EW_0_ (*r* = 0.187) and EW_−5_ (0.175) and the average weekly relative humidity at lags EW_0_ (*r* = 0.140), EW_−1_ (*r* = 0.130), EW_−2_ (*r* = 0.144), EW_−4_ (*r* = 0.139), EW_−5_ (*r* = 0.157), EW_−7_ (*r* = 0.176), EW_−8_ (*r* = 0.146), EW_−9_ (*r* = 0.155) and EW_−10_ (*r* = 0.158). However, no significant cross-correlation was detected between the weekly incidence of influenza cases and the average weekly ambient temperature at different lags. (Table 1).

**Table 1:** Cross-correlation between climatological parameters and weekly incidence of influenza cases, Abidjan, Côte d’Ivoire, 2007–2010

Climatological Parameters	Lags (Current and prior weeks)										
	**EW_0_	EW_−1_	EW_−2_	EW_−3_	EW_−4_	EW_−5_	EW_−6_	EW_−7_	EW_−8_	EW_−9_	EW_−10_
Rainfall	0.187*	0.067	0.040	0.104	0.034	0.175*	−0.007	−0.004	−0.069	−0.079	−0.006
Relative Humidity	0.141*	0.130*	0.144*	0.120	0.139*	0.157*	0.123	0.176*	0.146*	0.155*	0.158*
Ambient Temperature	−0.068	−0.025	−0.025	−0.033	−0.002	−0.035	0.007	0.004	−0.012	0.019	0.012

*Significant cross-correlation at *p* < 0.05

**EW denotes epidemiologic week; the subscripts indicate the number of weeks prior (0 being the same week, 1 the week prior, and so on)

We accepted 8 models after developing ARIMA univariate models. Among them, the ARIMA (2,0,0) had the best-fitted performance. We accepted 6 models by incorporating climatological variables specifically rainfall and relative humidity with various correlated lags in the ARIMAX multivariate modeling. Of the 6, the best fitting multivariate model (ARIMAX (2,0,0) _RF) included the number of influenza cases during 1-week and 2-weeks prior, and the rainfall during the current week and 5-weeks prior. The performance of this best fitting multivariate model showed an increase of >3 % for Akaike Information Criterion (AIC) and 2.5 % for Bayesian Information Criterion (BIC) compared to the best fitting univariate ARIMA (2,0,0) (Table 2).

**Table 2:** Univariate and multivariate ARIMA models of the weekly number of influenza cases and their performance, Abidjan, Cote d’Ivoire, 2007-2010

Models	Performance criteria		AR (*p*)	MA (*p*)	Breakpoint (*p*)	Rainfall (*RF*)		*Portmanteau test for residuals autocorrelation* ‘*white noise*’
	*AIC*	*BIC*	*Coef*.	*Coef*.	*Coef*.	*Lag* (*EW*_−*0*_)	*Lag* (*EW*_−*5*_)	*Q* (*p*)
*Univariate Arima models*								
ARIMA (0,0,2)	1305.828	1319.178	-	0.359 (*0.000*)	8.251 (*0.000*)	-	-	12.92 (*0.227*)
ARIMA (0,0,3)	1303.898	1320.586	-	0.127 (*0.017*)	8.382 (*0.000*)	-	-	8.25 (*0.604*)
ARIMA (0,0,5)	1301.275	1324.638	-	0.189 (*0.001*)	8.635 (*0.000*)	-	-	3.71 (*0.959*)
ARIMA (1,0,0)	1306.692	1316.705	0.506 (*0.000*)	-	8.440 (*0.000*)	-	-	16.07 (*0.097*)
ARIMA (1,0,1)	1298.622	1311.972	0.786 (*0.000*)	−0.374 (*0.000*)	9.326 (*0.000*)	-	-	2.377 (*0.667*)
ARIMA (1,0,3)	1296.892	1316.917	0.989 (*0.000*)	−0.242 (*0.000*)	16.602 (*0.000*)	-	-	6.626 (*0.760*)
ARIMA (2,0,0)	**1296.889**	**1310.24**	**0.238** (***0.000***)	-	**9.135** (***0.000***)	-	-	**5.502** (***0.855***)
ARIMA (2,0,5)	1302.240	1332.278	−0.526 (*0.004*)	0.156 (*0.035*)	8.837 (*0.000*)	-	-	1.851 (*0.997*)
*Multivariate Arimax models*								
ARIMAX (0,0,2) RF	1265.918	1285.797	-	0.332 (0.000)	6.467 (0.000)	0.02 (0.000)	0.023 (0.000)	13.08 (0.219)
ARIMAX (0,0,3) RF	1263.395	1286.588	-	0.137 (0.016)	6.542 (0.000)	0.021 (0.000)	0.023 (0.000)	8.246 (0.604)
ARIMAX (0,0,5) RF	1262.593	1292.412	-	0.149 (0.016)	6.905 (0.000)	0.018 (0.004)	0.025 (0.000)	4.359 (0.929)
ARIMAX (1,0,0) RF	1263.068	1279.634	0.521 (0.000)	-	6.572 (0.000)	0.018 (0.001)	0.028 (0.000)	15.21 (0.124)
ARIMAX (1,0,1) RF	1258.968	1278.847	0.736 (0.000)	−0.29 (0.002)	7.289 (0.000)	0.016 (0.007)	0.027 (0.000)	7.786 (0.649)
ARIMAX (2,0,0) RF	**1257.765**	**1277.644**	**0.192** (**0.001**)	-	**7.258** (**0.000**)	**0.017** (**0.005**)	**0.026** (**0.000**)	**5.894** (**0.824**)

Note: *AR* autoregressive coefficient, *MA* moving average coefficient, *Breakpoint* coefficient of the breakpoint variable: a surge in the dynamic of the incidence of influenza cases from week 19 of 2009 following the occurrence of pandemic Influenza 2009, installation of new sentinel sites and the acquisition of new diagnostic technique by the national influenza laboratory (National Influenza Centre: NIC, Pasteur Institute), *Q* coefficient of the Portmanteau test for residuals autocorrelation, *Coef*. coefficient, *p* *p*-value, *RF* rainfall, *ARIMA* (*X*) introduction of independent variables in the model (multivariate Arima), *AIC* akaïke information criterion, *BIC* Bayesian information criterion, *EW* epidemiologic week, the subscripts indicate the number of weeks prior (0 being the same week, 1 the week prior, and so on)

**Fig. 2:**
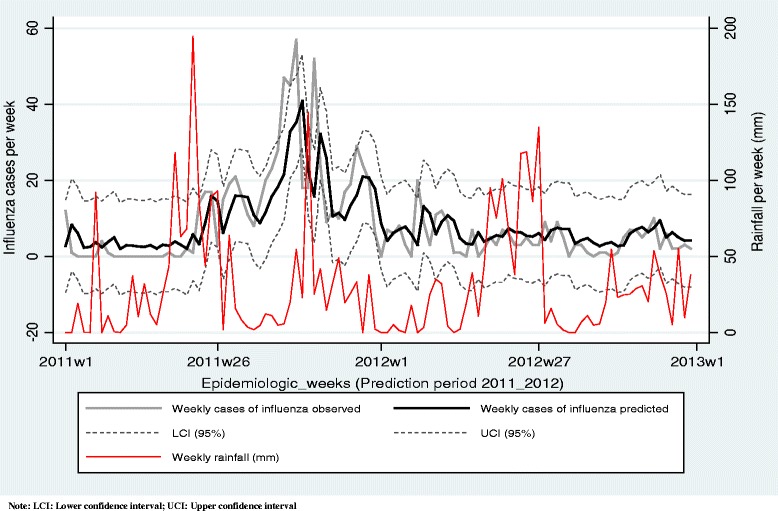
Weekly incidence of influenza cases observed and predicted over the period 2011EW1 to 2012EW52 with ARIMAX(2,0,0)RF by including rainfall, Abidjan, Cote d’Ivoire

The forecasting of the weekly incidence of influenza cases from 2011EW1 to 2012EW52 with ARIMAX (2,0,0) _RF by taking into account the observed values and the weekly-cumulated rainfall, permitted to predict the expected incidence of influenza cases during the following week. The observed numbers of influenza cases fell within the 95 % confidence interval of the predicted values in 97 of 104 weeks. However, the model ARIMAX (2,0,0) _RF has a large confidence interval which by default increase the likelihood that these observed values fall within the confidence bounds (Fig. 2).

## Discussion

By using ARIMA models, we explored the effects of climatological parameters on the occurrence of influenza cases in the city of Abidjan, which is located in a wet and rainy tropical region at 5.3°N. The addition of rainfall for the current week and for 5 weeks prior produced the best fitting and predicting model of the weekly incidence of influenza cases. This correlation found with rainfall at 5 weeks prior seems not like a simple correlation without biologic plausibility. In similar works in Hong Kong a subtropical city, Soebiyanto found a correlation between rainfall and influenza timing at 3 weeks prior [7] and at 3 weeks prior, and at 5 weeks-prior [15]. So, this could lead to in vivo experiment studies to explore this hypothesis.

Like ours, studies have shown that rainfall was a good predictor of influenza epidemic peaks in the tropical areas between the equator and the latitude 12.5° North/South [8]. Moreover, heavy rains coincide with the influenza epidemic peaks and rainfalls are significantly associated with high weekly incidence of influenza cases in Côte d’Ivoire [10, 11] and several other countries in the tropics [12, 16–18]. In some of these countries and cities near the equator like the city of Abidjan, the temperature does not fall below the threshold of 18–21 °C, so the seasonal influenza activity peaks occur during the months where the total rainfall is maximal and greater than 150 mm per month [8]. One other hypothesis is that rainfall leads people to spend more time indoors, thus increasing exposure to other people and this exposure increases the risk of acute respiratory infections [12]. This suggests that precipitation may allow increased exposure in overcrowded conditions and increase direct transmission or transmission by fomites [6–8, 12]. In fact, the climatological conditions of tropical rainy seasons may, encourage contact transmission of influenza virus, by increasing the amount of virus that is deposited on surfaces, and by increasing virus survival in droplets on surfaces. This evidence suggests that the increased incidence of influenza in tropical rainy seasons may be due to increased contact transmission [19]. So, the prediction of the expected number of influenza 1 week ahead can help clinicians in Abidjan to anticipate influenza epidemics during the rainy season. In these periods, health authorities can then reinforce influenza surveillance, further guide risk-communications to the public, foster influenza vaccine use among target populations and high-risk groups. These actions can reduce the burden of influenza on the population in cities like Abidjan.

In this study, relative humidity was poorly correlated with the weekly activity of influenza and was not a good predictor, because its values are moderately high and are between 54 % and 93 %. Even adjusted with rainfall, the effect of relative humidity on influenza transmission was non-significant. This lack of significant models with relative humidity, reveals that, although it is sometimes correlated with the occurrence of weekly influenza cases, its effect is very low or nonexistent. Furthermore Lowen et al., in an in vivo experiment study, argued that influenza transmission was completely blocked at a high relative humidity of 80 % [5]. However, relative humidity and precipitation are the best predictors of influenza epidemic peaks at low latitudes between the equator and 12.5° North/South [8]. Also, Soebiyanto et al. showed that rainfall and relative humidity were the best predictors of influenza transmission in Hong Kong, which has a subtropical location (22°N) [7]. But in reality, there is a U-shaped relationship between relative humidity and influenza virus survival, which was indicated by studies in vivo. Influenza transmission is high when the relative humidity is low or very high [20–22].

Despite the significantly and inversely correlation with the influenza activity in temperate zones, the ambient temperature was not correlated with the weekly incidence of influenza cases in our study. According to Tamerius et al., this parameter is not a significant predictor of influenza epidemic peaks between the equator and latitude 25° North/South [8]. The ambient temperature in Abidjan was very high and varied between 24.1 °C and 30.3 °C (temperature range = 6.2 °C). In this regard, Lowen et al., in vivo, stated that influenza transmission was high at low temperatures (5 °C) but was inefficient between 20 °C and 30 °C [5, 6]. But at such high temperatures, it has been shown that transmission through direct and indirect contact and short-range propagation of influenza predominated in the tropics [23]. The low outdoor temperatures in winter period promote indoor crowding, thereby increasing person-to-person contact rates [2, 24, 25]. It is also possible that even limited exposure to cold outdoor temperatures can have long-lasting physiological effects on hosts that make them more susceptible to infection or affect viral shedding [5].

Some potential limitations affected ours study. We modeled influenza timing in Abidjan only but not in several cities of the same climate zone. This is a challenge for the extrapolation of our findings to the south climate zone of Cote d’Ivoire in which Abidjan is located. Also, we found most moderate associations between rainfall and the detection of influenza. This limited added value of rainfall in the model and its predictive power should lead to study the effect of other potentially weather-related data such as atmospheric pressure, wind speed, absolute humidity, and solar radiation on the transmission of influenza. However, these climatologic factors data were not available for their assessment in the models. The continuation of this study will be helpful to deepen the effect of other climatologic factors adjusted to those already studied for determining their real impact on the influenza transmission in tropics. The breakpoint happened in the timing of influenza pandemic start at week 19 of 2009, since the beginning of the 2009 H1N1 pandemic led to the installation of new sentinel sites, and the acquisition of new real-time-PCR diagnosis techniques for the NIC. This resulted respectively to increased numbers of nasopharyngeal samples collected at the sentinel sites and positive strains isolated by the NIC. However, these events were corrected by introducing a breakpoint variable in the model.

## Conclusion

Based on the performance of the reference multivariate model, we determined the effects of rainfall in the modeling and forecasting of weekly incidence of influenza cases in Abidjan. Prevention and treatment strategies that could be implemented to anticipate epidemics immediately before the start of the influenza season are vaccination of the population particularly of high-risk groups, sensitization of clinicians and communication campaigns within the communities, provision of stocks of antivirals. The implementation of these measures can help to mitigate the effects of influenza on the population of this tropical city.

## Additional files

Additional file 1: Data correlation test and ARIMA model fitting. (DOCX 19 kb) Additional file 2: Data for multivariate ARIMAX forecasting. (DOCX 29 kb)

## Abbreviations

AIC: Akaike information criterion
AR: Autoregressive
ARIMA (X: Explicative): Autoregressive integrated moving average
CDC: Centers for disease control and prevention
Coef.: Coefficient
EW: Epidemiologic week
INHP: National institute of public hygiene
LCI: Lower confidence interval
MA: Moving average
MDCK: Madin-darby canine kidney
NIC: National influenza centre
p: *p*-value
Q: Coefficient of the portmanteau test
Rel. Hum.: Relative humidity
RF: Rainfall
RT-PCR: Retro transcriptase polymerase chain reaction
SARI: Severe acute respiratory infections
SODEXAM: Airport operating development aviation and meteorology company
Temp.: Ambient temperature
UCI: Upper confidence interval
WHO: World health organization
