# Supplementary material for: Effects of climatological parameters in modeling and forecasting seasonal influenza transmission in Abidjan, Cote d’Ivoire
Source: BMC Public Health. 2016 Sep 13;16(1):972. doi: 10.1186/s12889-016-3503-1 (PMC5022141; doi:10.1186/s12889-016-3503-1)
Supplement: Additional file 1: — Data correlation test and ARIMA model fitting. (DOCX 19 kb) [file 12889_2016_3503_MOESM1_ESM.docx]

**Additional file1: Data from 2007 to 2010 used for correlation test between climatological parameters and seasonal influenza and for ARIMA model fitting**

| Epidemiologic_week | Influenza cases | BreakPoint_Variable | Rainfall (mm) | Relative Humidity (%) | Ambient Temperature (°C) |
| --- | --- | --- | --- | --- | --- |
| 2007w1 | 0 | 0 | 0 | 54 | 25,3 |
| 2007w2 | 0 | 0 | 0 | 83 | 26,6 |
| 2007w3 | 0 | 0 | 0 | 77 | 26,5 |
| 2007w4 | 0 | 0 | 0 | 83 | 29,1 |
| 2007w5 | 0 | 0 | 40 | 84 | 29,3 |
| 2007w6 | 0 | 0 | 0 | 85 | 28,7 |
| 2007w7 | 0 | 0 | 2.3 | 86 | 28,6 |
| 2007w8 | 0 | 0 | 43 | 85 | 28,5 |
| 2007w9 | 0 | 0 | 30.3 | 85 | 28,8 |
| 2007w10 | 0 | 0 | 32.8 | 82 | 29,4 |
| 2007w11 | 1 | 0 | 43.5 | 83 | 29 |
| 2007w12 | 1 | 0 | 6.1 | 83 | 28,7 |
| 2007w13 | 0 | 0 | 30.1 | 85 | 28,2 |
| 2007w14 | 1 | 0 | 47.6 | 85 | 28,1 |
| 2007w15 | 8 | 0 | 0 | 84 | 29,6 |
| 2007w16 | 7 | 0 | 62 | 86 | 28,9 |
| 2007w17 | 9 | 0 | 5.8 | 87 | 27,7 |
| 2007w18 | 3 | 0 | 5.5 | 90 | 27,8 |
| 2007w19 | 9 | 0 | 25 | 86 | 28,5 |
| 2007w20 | 6 | 0 | 15 | 86 | 28,1 |
| 2007w21 | 3 | 0 | 69.7 | 83 | 28,5 |
| 2007w22 | 3 | 0 | 37.1 | 85 | 27,4 |
| 2007w23 | 14 | 0 | 190.8 | 88 | 26,4 |
| 2007w24 | 6 | 0 | 47.6 | 86 | 26,6 |
| 2007w25 | 0 | 0 | 13.6 | 83 | 27,6 |
| 2007w26 | 1 | 0 | 12.2 | 87 | 26,5 |
| 2007w27 | 1 | 0 | 103.1 | 86 | 26,4 |
| 2007w28 | 0 | 0 | .2 | 84 | 27,1 |
| 2007w29 | 2 | 0 | .1 | 89 | 25,8 |
| 2007w30 | 4 | 0 | 82.5 | 91 | 25,5 |
| 2007w31 | 1 | 0 | 3.4 | 87 | 25,4 |
| 2007w32 | 0 | 0 | 3.5 | 92 | 24,7 |
| 2007w33 | 0 | 0 | .9 | 92 | 24,1 |
| 2007w34 | 0 | 0 | 2.6 | 91 | 25 |
| 2007w35 | 1 | 0 | 35.2 | 91 | 25,1 |
| 2007w36 | 0 | 0 | 2.7 | 91 | 24,6 |
| 2007w37 | 0 | 0 | 0 | 90 | 25,8 |
| 2007w38 | 3 | 0 | 29.8 | 92 | 26 |
| 2007w39 | 4 | 0 | 4.5 | 87 | 26,7 |
| 2007w40 | 0 | 0 | 66.4 | 87 | 26,6 |
| 2007w41 | 0 | 0 | 41.4 | 88 | 26,1 |
| 2007w42 | 0 | 0 | 50.6 | 85 | 27,5 |
| 2007w43 | 0 | 0 | 73.6 | 85 | 27 |
| 2007w44 | 0 | 0 | 56.8 | 83 | 27,7 |
| 2007w45 | 1 | 0 | 17.4 | 82 | 27,7 |
| 2007w46 | 0 | 0 | 66.9 | 84 | 28,1 |
| 2007w47 | 0 | 0 | 14.2 | 80 | 28,8 |
| 2007w48 | 0 | 0 | 28.6 | 80 | 29,2 |
| 2007w49 | 0 | 0 | 29.4 | 77 | 27,8 |
| 2007w50 | 0 | 0 | 21.9 | 82 | 28,6 |
| 2007w51 | 1 | 0 | 37.1 | 83 | 28 |
| 2007w52 | 0 | 0 | 56.7 | 84 | 28,4 |
| 2008w1 | 0 | 0 | 0 | 78 | 27,7 |
| 2008w2 | 0 | 0 | 0 | 67 | 26 |
| 2008w3 | 0 | 0 | 24 | 86 | 27 |
| 2008w4 | 0 | 0 | 0 | 75 | 26,1 |
| 2008w5 | 0 | 0 | 0 | 71 | 26,5 |
| 2008w6 | 0 | 0 | 0 | 81 | 29,1 |
| 2008w7 | 0 | 0 | .1 | 79 | 29,9 |
| 2008w8 | 0 | 0 | .7 | 79 | 29,9 |
| 2008w9 | 1 | 0 | 90.5 | 79 | 28,6 |
| 2008w10 | 0 | 0 | 17.1 | 83 | 28,6 |
| 2008w11 | 0 | 0 | 3.3 | 86 | 27,8 |
| 2008w12 | 0 | 0 | 3 | 86 | 28,1 |
| 2008w13 | 0 | 0 | 1.1 | 85 | 28,4 |
| 2008w14 | 0 | 0 | 53.7 | 85 | 28,1 |
| 2008w15 | 2 | 0 | 14.8 | 83 | 29,1 |
| 2008w16 | 5 | 0 | 7.7 | 80 | 29,3 |
| 2008w17 | 4 | 0 | 68.4 | 82 | 28,4 |
| 2008w18 | 4 | 0 | .8 | 81 | 28,4 |
| 2008w19 | 4 | 0 | 60.9 | 83 | 28,5 |
| 2008w20 | 3 | 0 | 193 | 84 | 27,9 |
| 2008w21 | 0 | 0 | 154.3 | 83 | 27,7 |
| 2008w22 | 1 | 0 | 100 | 87 | 26,8 |
| 2008w23 | 1 | 0 | 158.6 | 89 | 27,2 |
| 2008w24 | 1 | 0 | 33.7 | 84 | 27,6 |
| 2008w25 | 0 | 0 | 190.5 | 86 | 26,8 |
| 2008w26 | 1 | 0 | 92.1 | 86 | 27,4 |
| 2008w27 | 0 | 0 | 10.5 | 88 | 26,7 |
| 2008w28 | 0 | 0 | .4 | 83 | 27,4 |
| 2008w29 | 2 | 0 | 22.5 | 86 | 26,9 |
| 2008w30 | 3 | 0 | 89.5 | 91 | 25,5 |
| 2008w31 | 1 | 0 | 0 | 87 | 26,4 |
| 2008w32 | 2 | 0 | 0 | 86 | 26,3 |
| 2008w33 | 4 | 0 | 3.5 | 89 | 25,2 |
| 2008w34 | 6 | 0 | 0 | 89 | 24,6 |
| 2008w35 | 2 | 0 | 8.2 | 91 | 24,6 |
| 2008w36 | 0 | 0 | .9 | 89 | 24,8 |
| 2008w37 | 3 | 0 | 0 | 90 | 25,6 |
| 2008w38 | 1 | 0 | 43.8 | 90 | 25,4 |
| 2008w39 | 1 | 0 | 2 | 90 | 25,7 |
| 2008w40 | 1 | 0 | .2 | 88 | 26,3 |
| 2008w41 | 3 | 0 | 13.8 | 87 | 26,6 |
| 2008w42 | 0 | 0 | 8.2 | 85 | 27,4 |
| 2008w43 | 0 | 0 | 5.3 | 82 | 28,2 |
| 2008w44 | 1 | 0 | 36.3 | 83 | 28,3 |
| 2008w45 | 0 | 0 | 98.5 | 84 | 28 |
| 2008w46 | 1 | 0 | 22.5 | 82 | 28 |
| 2008w47 | 0 | 0 | 33.2 | 81 | 29,2 |
| 2008w48 | 9 | 0 | 10 | 80 | 29,5 |
| 2008w49 | 5 | 0 | 29 | 83 | 27,8 |
| 2008w50 | 5 | 0 | 4.8 | 82 | 28,2 |
| 2008w51 | 0 | 0 | .2 | 83 | 28,9 |
| 2008w52 | 1 | 0 | .1 | 82 | 29,4 |
| 2009w1 | 0 | 0 | 15.9 | 84 | 28,9 |
| 2009w2 | 2 | 0 | 3.8 | 84 | 28,4 |
| 2009w3 | 4 | 0 | 0 | 86 | 28,8 |
| 2009w4 | 4 | 0 | 0 | 58 | 25,7 |
| 2009w5 | 4 | 0 | .6 | 86 | 27,4 |
| 2009w6 | 6 | 0 | 0 | 86 | 28,5 |
| 2009w7 | 9 | 0 | 68.3 | 86 | 27,6 |
| 2009w8 | 5 | 0 | 0 | 88 | 28,1 |
| 2009w9 | 7 | 0 | 19.7 | 88 | 27,7 |
| 2009w10 | 7 | 0 | 33.9 | 89 | 28,3 |
| 2009w11 | 1 | 0 | 11.9 | 88 | 28,1 |
| 2009w12 | 6 | 0 | 30.4 | 89 | 28 |
| 2009w13 | 2 | 0 | .2 | 87 | 28,3 |
| 2009w14 | 2 | 0 | 0 | 87 | 27,5 |
| 2009w15 | 5 | 0 | 12.4 | 88 | 27,9 |
| 2009w16 | 2 | 0 | 28.9 | 87 | 27,2 |
| 2009w17 | 6 | 0 | 22.9 | 84 | 28,8 |
| 2009w18 | 1 | 0 | 1.8 | 85 | 29,2 |
| 2009w19 | 35 | 1 | 206.2 | 87 | 27,3 |
| 2009w20 | 31 | 1 | 24.5 | 86 | 28,3 |
| 2009w21 | 20 | 1 | 6.3 | 82 | 29,4 |
| 2009w22 | 27 | 1 | 8.3 | 83 | 28,6 |
| 2009w23 | 8 | 1 | 116.6 | 83 | 28,5 |
| 2009w24 | 29 | 1 | 281.9 | 85 | 27,6 |
| 2009w25 | 6 | 1 | 193.5 | 87 | 26,9 |
| 2009w26 | 8 | 1 | 139.5 | 84 | 27,1 |
| 2009w27 | 11 | 1 | 22.6 | 86 | 26,2 |
| 2009w28 | 2 | 1 | 63.7 | 88 | 25,6 |
| 2009w29 | 10 | 1 | 20.1 | 83 | 26,6 |
| 2009w30 | 30 | 1 | 10.6 | 88 | 25,6 |
| 2009w31 | 3 | 1 | 8.3 | 91 | 24,6 |
| 2009w32 | 3 | 1 | 4 | 92 | 24,6 |
| 2009w33 | 4 | 1 | 2.4 | 91 | 24,5 |
| 2009w34 | 10 | 1 | 2.3 | 91 | 24,5 |
| 2009w35 | 23 | 1 | 0 | 93 | 24,4 |
| 2009w36 | 14 | 1 | .5 | 92 | 24,8 |
| 2009w37 | 6 | 1 | 21.9 | 93 | 24,3 |
| 2009w38 | 13 | 1 | .1 | 92 | 24,5 |
| 2009w39 | 23 | 1 | 1.4 | 91 | 25,3 |
| 2009w40 | 21 | 1 | .5 | 92 | 25,6 |
| 2009w41 | 14 | 1 | .4 | 90 | 25,5 |
| 2009w42 | 22 | 1 | 1.3 | 89 | 26 |
| 2009w43 | 6 | 1 | .2 | 89 | 26,7 |
| 2009w44 | 4 | 1 | 1.1 | 87 | 27,4 |
| 2009w45 | 4 | 1 | 6.7 | 84 | 28,2 |
| 2009w46 | 2 | 1 | 53.1 | 81 | 28,4 |
| 2009w47 | 0 | 1 | 3 | 83 | 28,8 |
| 2009w48 | 2 | 1 | 18.4 | 85 | 28,9 |
| 2009w49 | 1 | 1 | .3 | 80 | 29,3 |
| 2009w50 | 9 | 1 | 46.6 | 81 | 28,4 |
| 2009w51 | 1 | 1 | .2 | 80 | 29,4 |
| 2009w52 | 0 | 1 | 93.6 | 85 | 28,9 |
| 2010w1 | 0 | 1 | .3 | 88 | 28,8 |
| 2010w2 | 9 | 1 | 45.9 | 87 | 28,6 |
| 2010w3 | 8 | 1 | 0 | 89 | 28 |
| 2010w4 | 5 | 1 | .6 | 88 | 28,6 |
| 2010w5 | 3 | 1 | 13 | 85 | 28,9 |
| 2010w6 | 8 | 1 | 0 | 84 | 29,7 |
| 2010w7 | 4 | 1 | 40.2 | 83 | 29,2 |
| 2010w8 | 3 | 1 | 0 | 83 | 29,4 |
| 2010w9 | 1 | 1 | 0 | 84 | 29,8 |
| 2010w10 | 1 | 1 | 0 | 85 | 30,2 |
| 2010w11 | 1 | 1 | 69.7 | 82 | 29,6 |
| 2010w12 | 12 | 1 | 18.3 | 82 | 28,6 |
| 2010w13 | 6 | 1 | 9.4 | 83 | 30,1 |
| 2010w14 | 9 | 1 | 26.4 | 82 | 30,3 |
| 2010w15 | 2 | 1 | 20.2 | 85 | 29,3 |
| 2010w16 | 1 | 1 | .2 | 83 | 30,3 |
| 2010w17 | 0 | 1 | 34.3 | 83 | 29 |
| 2010w18 | 2 | 1 | 165.2 | 83 | 29,1 |
| 2010w19 | 0 | 1 | 16.3 | 84 | 29 |
| 2010w20 | 13 | 1 | 20 | 82 | 29,6 |
| 2010w21 | 13 | 1 | 206.2 | 83 | 28,5 |
| 2010w22 | 6 | 1 | 122.2 | 84 | 28,5 |
| 2010w23 | 6 | 1 | 59.7 | 86 | 27,5 |
| 2010w24 | 16 | 1 | 107.2 | 88 | 27,2 |
| 2010w25 | 14 | 1 | 96.7 | 86 | 27,3 |
| 2010w26 | 19 | 1 | 128.1 | 86 | 27,3 |
| 2010w27 | 2 | 1 | 11.7 | 84 | 27 |
| 2010w28 | 0 | 1 | 190.6 | 91 | 25,9 |
| 2010w29 | 2 | 1 | 20.6 | 88 | 26,2 |
| 2010w30 | 0 | 1 | .2 | 84 | 26,1 |
| 2010w31 | 0 | 1 | 2.1 | 89 | 25,3 |
| 2010w32 | 0 | 1 | 0 | 92 | 24,7 |
| 2010w33 | 11 | 1 | 11.3 | 91 | 24,5 |
| 2010w34 | 1 | 1 | 43.8 | 90 | 24,5 |
| 2010w35 | 0 | 1 | .7 | 92 | 24,4 |
| 2010w36 | 0 | 1 | 15.2 | 93 | 25,1 |
| 2010w37 | 0 | 1 | 1 | 92 | 25,1 |
| 2010w38 | 0 | 1 | 5.4 | 89 | 25,9 |
| 2010w39 | 0 | 1 | 210.9 | 90 | 26,1 |
| 2010w40 | 0 | 1 | 25.3 | 86 | 26,9 |
| 2010w41 | 1 | 1 | 42.9 | 86 | 27,2 |
| 2010w42 | 10 | 1 | 40.4 | 86 | 27,2 |
| 2010w43 | 12 | 1 | 26.2 | 85 | 27,6 |
| 2010w44 | 38 | 1 | 28.2 | 82 | 28 |
| 2010w45 | 13 | 1 | 29.8 | 83 | 27,7 |
| 2010w46 | 21 | 1 | 29.9 | 84 | 27,7 |
| 2010w47 | 2 | 1 | 40.2 | 81 | 28,1 |
| 2010w48 | 3 | 1 | 45.8 | 81 | 28,3 |
| 2010w49 | 0 | 1 | 40.3 | 80 | 28,9 |
| 2010w50 | 1 | 1 | 27.9 | 81 | 28 |
| 2010w51 | 0 | 1 | .8 | 80 | 29,1 |
| 2010w52 | 0 | 1 | 57.7 | 79 | 29,5 |
