# Supplementary material for: Effects of climatological parameters in modeling and forecasting seasonal influenza transmission in Abidjan, Cote d’Ivoire
Source: BMC Public Health. 2016 Sep 13;16(1):972. doi: 10.1186/s12889-016-3503-1 (PMC5022141; doi:10.1186/s12889-016-3503-1)
Supplement: Additional file 2: — Data for multivariate ARIMAX forecasting. (DOCX 29 kb) [file 12889_2016_3503_MOESM2_ESM.docx]

**Additional file2: Data from 2007 to 2012 used for ARIMAX model forecasting.**

| Epidemiologic_week | Influenza cases | BreakPoint_Variable | Rainfall (mm) | yhat | sigma | UCI | LCI |
| --- | --- | --- | --- | --- | --- | --- | --- |
| 2007w1 | 0 | 0 | 0 | . | 68.68825 | . | . |
| 2007w2 | 0 | 0 | 0 | . | 68.68825 | . | . |
| 2007w3 | 0 | 0 | 0 | . | 68.68825 | . | . |
| 2007w4 | 0 | 0 | 0 | . | 68.68825 | . | . |
| 2007w5 | 0 | 0 | 40 | . | 68.68825 | . | . |
| 2007w6 | 0 | 0 | 0 | .1695597 | 68.68825 | 16.41372 | -16.0746 |
| 2007w7 | 0 | 0 | 2.3 | -.4288372 | 39.7711 | 11.93177 | -12.78945 |
| 2007w8 | 0 | 0 | 43 | .871786 | 38.64103 | 13.05552 | -11.31195 |
| 2007w9 | 0 | 0 | 30.3 | -.0785553 | 38.64103 | 12.10518 | -12.26229 |
| 2007w10 | 0 | 0 | 32.8 | -.1566008 | 38.64103 | 12.02713 | -12.34034 |
| 2007w11 | 1 | 0 | 43.5 | .5193383 | 38.64103 | 12.70307 | -11.6644 |
| 2007w12 | 1 | 0 | 6.1 | .223235 | 38.64103 | 12.40697 | -11.9605 |
| 2007w13 | 0 | 0 | 30.1 | .6923802 | 38.64103 | 12.87611 | -11.49135 |
| 2007w14 | 1 | 0 | 47.6 | .8551007 | 38.64103 | 13.03884 | -11.32863 |
| 2007w15 | 8 | 0 | 0 | -.0425272 | 38.64103 | 12.14121 | -12.22626 |
| 2007w16 | 7 | 0 | 62 | 4.852875 | 38.64103 | 17.03661 | -7.33086 |
| 2007w17 | 9 | 0 | 5.8 | 5.525506 | 38.64103 | 17.70924 | -6.658228 |
| 2007w18 | 3 | 0 | 5.5 | 5.01625 | 38.64103 | 17.19998 | -7.167485 |
| 2007w19 | 9 | 0 | 25 | 4.135141 | 38.64103 | 16.31888 | -8.048594 |
| 2007w20 | 6 | 0 | 15 | 5.034558 | 38.64103 | 17.21829 | -7.149177 |
| 2007w21 | 3 | 0 | 69.7 | 5.178863 | 38.64103 | 17.3626 | -7.004872 |
| 2007w22 | 3 | 0 | 37.1 | 3.030696 | 38.64103 | 15.21443 | -9.153039 |
| 2007w23 | 14 | 0 | 190.8 | 2.93715 | 38.64103 | 15.12088 | -9.246585 |
| 2007w24 | 6 | 0 | 47.6 | 8.716496 | 38.64103 | 20.90023 | -3.467238 |
| 2007w25 | 0 | 0 | 13.6 | 3.328413 | 38.64103 | 15.51215 | -8.855322 |
| 2007w26 | 1 | 0 | 12.2 | 2.727882 | 38.64103 | 14.91162 | -9.455853 |
| 2007w27 | 1 | 0 | 103.1 | .5339221 | 38.64103 | 12.71766 | -11.64981 |
| 2007w28 | 0 | 0 | .2 | .6477994 | 38.64103 | 12.83153 | -11.53594 |
| 2007w29 | 2 | 0 | .1 | -.9188409 | 38.64103 | 11.26489 | -13.10258 |
| 2007w30 | 4 | 0 | 82.5 | 2.994591 | 38.64103 | 15.17833 | -9.189143 |
| 2007w31 | 1 | 0 | 3.4 | 1.739431 | 38.64103 | 13.92317 | -10.4443 |
| 2007w32 | 0 | 0 | 3.5 | .4087999 | 38.64103 | 12.59254 | -11.77493 |
| 2007w33 | 0 | 0 | .9 | 1.175203 | 38.64103 | 13.35894 | -11.00853 |
| 2007w34 | 0 | 0 | 2.6 | -.602111 | 38.64103 | 11.58162 | -12.78585 |
| 2007w35 | 1 | 0 | 35.2 | .4570569 | 38.64103 | 12.64079 | -11.72668 |
| 2007w36 | 0 | 0 | 2.7 | .5253223 | 38.64103 | 12.70906 | -11.65841 |
| 2007w37 | 0 | 0 | 0 | -.2436476 | 38.64103 | 11.94009 | -12.42738 |
| 2007w38 | 3 | 0 | 29.8 | .6469402 | 38.64103 | 12.83068 | -11.53679 |
| 2007w39 | 4 | 0 | 4.5 | 1.420132 | 38.64103 | 13.60387 | -10.7636 |
| 2007w40 | 0 | 0 | 66.4 | 2.922754 | 38.64103 | 15.10649 | -9.260981 |
| 2007w41 | 0 | 0 | 41.4 | 1.330766 | 38.64103 | 13.5145 | -10.85297 |
| 2007w42 | 0 | 0 | 50.6 | -.5574504 | 38.64103 | 11.62628 | -12.74119 |
| 2007w43 | 0 | 0 | 73.6 | .9522881 | 38.64103 | 13.13602 | -11.23145 |
| 2007w44 | 0 | 0 | 56.8 | -.1212802 | 38.64103 | 12.06245 | -12.30501 |
| 2007w45 | 1 | 0 | 17.4 | -.3491549 | 38.64103 | 11.83458 | -12.53289 |
| 2007w46 | 0 | 0 | 66.9 | 1.046322 | 38.64103 | 13.23006 | -11.13741 |
| 2007w47 | 0 | 0 | 14.2 | .290538 | 38.64103 | 12.47427 | -11.8932 |
| 2007w48 | 0 | 0 | 28.6 | -.6467872 | 38.64103 | 11.53695 | -12.83052 |
| 2007w49 | 0 | 0 | 29.4 | .8760011 | 38.64103 | 13.05974 | -11.30773 |
| 2007w50 | 0 | 0 | 21.9 | -.4574943 | 38.64103 | 11.72624 | -12.64123 |
| 2007w51 | 1 | 0 | 37.1 | .3142242 | 38.64103 | 12.49796 | -11.86951 |
| 2007w52 | 0 | 0 | 56.7 | .9426603 | 38.64103 | 13.1264 | -11.24107 |
| 2008w1 | 0 | 0 | 0 | -.219635 | 38.64103 | 11.9641 | -12.40337 |
| 2008w2 | 0 | 0 | 0 | -.3432429 | 38.64103 | 11.84049 | -12.52698 |
| 2008w3 | 0 | 0 | 24 | .678224 | 38.64103 | 12.86196 | -11.50551 |
| 2008w4 | 0 | 0 | 0 | -.3664715 | 38.64103 | 11.81726 | -12.55021 |
| 2008w5 | 0 | 0 | 0 | -.190946 | 38.64103 | 11.99279 | -12.37468 |
| 2008w6 | 0 | 0 | 0 | .3053694 | 38.64103 | 12.4891 | -11.87837 |
| 2008w7 | 0 | 0 | .1 | -.1844242 | 38.64103 | 11.99931 | -12.36816 |
| 2008w8 | 0 | 0 | .7 | .0470578 | 38.64103 | 12.23079 | -12.13668 |
| 2008w9 | 1 | 0 | 90.5 | .8033088 | 38.64103 | 12.98704 | -11.38043 |
| 2008w10 | 0 | 0 | 17.1 | .6323558 | 38.64103 | 12.81609 | -11.55138 |
| 2008w11 | 0 | 0 | 3.3 | -.9121923 | 38.64103 | 11.27154 | -13.09593 |
| 2008w12 | 0 | 0 | 3 | .9267399 | 38.64103 | 13.11047 | -11.257 |
| 2008w13 | 0 | 0 | 1.1 | -.4883147 | 38.64103 | 11.69542 | -12.67205 |
| 2008w14 | 0 | 0 | 53.7 | .505577 | 38.64103 | 12.68931 | -11.67816 |
| 2008w15 | 2 | 0 | 14.8 | .1540866 | 38.64103 | 12.33782 | -12.02965 |
| 2008w16 | 5 | 0 | 7.7 | .4697174 | 38.64103 | 12.65345 | -11.71402 |
| 2008w17 | 4 | 0 | 68.4 | 4.110684 | 38.64103 | 16.29442 | -8.07305 |
| 2008w18 | 4 | 0 | .8 | 2.665963 | 38.64103 | 14.8497 | -9.517772 |
| 2008w19 | 4 | 0 | 60.9 | 2.608285 | 38.64103 | 14.79202 | -9.57545 |
| 2008w20 | 3 | 0 | 193 | 5.319783 | 38.64103 | 17.50352 | -6.863952 |
| 2008w21 | 0 | 0 | 154.3 | 2.321614 | 38.64103 | 14.50535 | -9.862122 |
| 2008w22 | 1 | 0 | 100 | -.1950971 | 38.64103 | 11.98864 | -12.37883 |
| 2008w23 | 1 | 0 | 158.6 | 1.945695 | 38.64103 | 14.12943 | -10.23804 |
| 2008w24 | 1 | 0 | 33.7 | .2702767 | 38.64103 | 12.45401 | -11.91346 |
| 2008w25 | 0 | 0 | 190.5 | .867628 | 38.64103 | 13.05136 | -11.31611 |
| 2008w26 | 1 | 0 | 92.1 | 1.991425 | 38.64103 | 14.17516 | -10.19231 |
| 2008w27 | 0 | 0 | 10.5 | -2.322668 | 38.64103 | 9.861067 | -14.5064 |
| 2008w28 | 0 | 0 | .4 | 1.4137 | 38.64103 | 13.59743 | -10.77003 |
| 2008w29 | 2 | 0 | 22.5 | -.1166216 | 38.64103 | 12.06711 | -12.30036 |
| 2008w30 | 3 | 0 | 89.5 | 1.612604 | 38.64103 | 13.79634 | -10.57113 |
| 2008w31 | 1 | 0 | 0 | 1.761777 | 38.64103 | 13.94551 | -10.42196 |
| 2008w32 | 2 | 0 | 0 | .2375648 | 38.64103 | 12.4213 | -11.94617 |
| 2008w33 | 4 | 0 | 3.5 | 2.206645 | 38.64103 | 14.39038 | -9.977089 |
| 2008w34 | 6 | 0 | 0 | 1.869323 | 38.64103 | 14.05306 | -10.31441 |
| 2008w35 | 2 | 0 | 8.2 | 4.098921 | 38.64103 | 16.28266 | -8.084814 |
| 2008w36 | 0 | 0 | .9 | 2.156969 | 38.64103 | 14.3407 | -10.02677 |
| 2008w37 | 3 | 0 | 0 | .1975574 | 38.64103 | 12.38129 | -11.98618 |
| 2008w38 | 1 | 0 | 43.8 | 2.101072 | 38.64103 | 14.28481 | -10.08266 |
| 2008w39 | 1 | 0 | 2 | .991401 | 38.64103 | 13.17514 | -11.19233 |
| 2008w40 | 1 | 0 | .2 | .1789334 | 38.64103 | 12.36267 | -12.0048 |
| 2008w41 | 3 | 0 | 13.8 | 1.349223 | 38.64103 | 13.53296 | -10.83451 |
| 2008w42 | 0 | 0 | 8.2 | 1.550016 | 38.64103 | 13.73375 | -10.63372 |
| 2008w43 | 0 | 0 | 5.3 | .4413784 | 38.64103 | 12.62511 | -11.74236 |
| 2008w44 | 1 | 0 | 36.3 | .4005856 | 38.64103 | 12.58432 | -11.78315 |
| 2008w45 | 0 | 0 | 98.5 | 1.318774 | 38.64103 | 13.50251 | -10.86496 |
| 2008w46 | 1 | 0 | 22.5 | -.1009138 | 38.64103 | 12.08282 | -12.28465 |
| 2008w47 | 0 | 0 | 33.2 | .0400683 | 38.64103 | 12.2238 | -12.14367 |
| 2008w48 | 9 | 0 | 10 | .9201577 | 38.64103 | 13.10389 | -11.26358 |
| 2008w49 | 5 | 0 | 29 | 4.311552 | 38.64103 | 16.49529 | -7.872183 |
| 2008w50 | 5 | 0 | 4.8 | 4.539147 | 38.64103 | 16.72288 | -7.644588 |
| 2008w51 | 0 | 0 | .2 | 3.120854 | 38.64103 | 15.30459 | -9.062881 |
| 2008w52 | 1 | 0 | .1 | 1.117064 | 38.64103 | 13.3008 | -11.06667 |
| 2009w1 | 0 | 0 | 15.9 | .5447676 | 38.64103 | 12.7285 | -11.63897 |
| 2009w2 | 2 | 0 | 3.8 | .2068884 | 38.64103 | 12.39062 | -11.97685 |
| 2009w3 | 4 | 0 | 0 | .8961074 | 38.64103 | 13.07984 | -11.28763 |
| 2009w4 | 4 | 0 | 0 | 2.64129 | 38.64103 | 14.82502 | -9.542445 |
| 2009w5 | 4 | 0 | .6 | 2.766443 | 38.64103 | 14.95018 | -9.417292 |
| 2009w6 | 6 | 0 | 0 | 2.830872 | 38.64103 | 15.01461 | -9.352862 |
| 2009w7 | 9 | 0 | 68.3 | 4.517062 | 38.64103 | 16.7008 | -7.666673 |
| 2009w8 | 5 | 0 | 0 | 5.837034 | 38.64103 | 18.02077 | -6.346701 |
| 2009w9 | 7 | 0 | 19.7 | 3.546469 | 38.64103 | 15.7302 | -8.637265 |
| 2009w10 | 7 | 0 | 33.9 | 5.751641 | 38.64103 | 17.93538 | -6.432094 |
| 2009w11 | 1 | 0 | 11.9 | 4.298036 | 38.64103 | 16.48177 | -7.885699 |
| 2009w12 | 6 | 0 | 30.4 | 1.925591 | 38.64103 | 14.10933 | -10.25814 |
| 2009w13 | 2 | 0 | .2 | 3.535789 | 38.64103 | 15.71952 | -8.647946 |
| 2009w14 | 2 | 0 | 0 | 1.638093 | 38.64103 | 13.82183 | -10.54564 |
| 2009w15 | 5 | 0 | 12.4 | 1.873118 | 38.64103 | 14.05685 | -10.31062 |
| 2009w16 | 2 | 0 | 28.9 | 3.08726 | 38.64103 | 15.271 | -9.096475 |
| 2009w17 | 6 | 0 | 22.9 | 2.005433 | 38.64103 | 14.18917 | -10.1783 |
| 2009w18 | 1 | 0 | 1.8 | 3.3832 | 38.64103 | 15.56694 | -8.800534 |
| 2009w19 | 35 | 1 | 206.2 | 12.19769 | 38.64103 | 24.38143 | .0139581 |
| 2009w20 | 31 | 1 | 24.5 | 23.30136 | 38.64103 | 35.4851 | 11.11763 |
| 2009w21 | 20 | 1 | 6.3 | 22.62848 | 38.64103 | 34.81221 | 10.44474 |
| 2009w22 | 27 | 1 | 8.3 | 20.94323 | 38.64103 | 33.12697 | 8.759499 |
| 2009w23 | 8 | 1 | 116.6 | 20.25235 | 38.64103 | 32.43608 | 8.068611 |
| 2009w24 | 29 | 1 | 281.9 | 14.02135 | 38.64103 | 26.20508 | 1.837614 |
| 2009w25 | 6 | 1 | 193.5 | 19.85871 | 38.64103 | 32.04244 | 7.674971 |
| 2009w26 | 8 | 1 | 139.5 | 9.749427 | 38.64103 | 21.93316 | -2.434308 |
| 2009w27 | 11 | 1 | 22.6 | 8.295329 | 38.64103 | 20.47906 | -3.888406 |
| 2009w28 | 2 | 1 | 63.7 | 9.213011 | 38.64103 | 21.39675 | -2.970724 |
| 2009w29 | 10 | 1 | 20.1 | 6.185547 | 38.64103 | 18.36928 | -5.998188 |
| 2009w30 | 30 | 1 | 10.6 | 7.252318 | 38.64103 | 19.43605 | -4.931417 |
| 2009w31 | 3 | 1 | 8.3 | 21.14077 | 38.64103 | 33.32451 | 8.957039 |
| 2009w32 | 3 | 1 | 4 | 8.984139 | 38.64103 | 21.16787 | -3.199595 |
| 2009w33 | 4 | 1 | 2.4 | 4.70165 | 38.64103 | 16.88538 | -7.482085 |
| 2009w34 | 10 | 1 | 2.3 | 5.28844 | 38.64103 | 17.47217 | -6.895295 |
| 2009w35 | 23 | 1 | 0 | 8.623806 | 38.64103 | 20.80754 | -3.559929 |
| 2009w36 | 14 | 1 | .5 | 16.66698 | 38.64103 | 28.85071 | 4.483244 |
| 2009w37 | 6 | 1 | 21.9 | 14.22393 | 38.64103 | 26.40766 | 2.040195 |
| 2009w38 | 13 | 1 | .1 | 8.150264 | 38.64103 | 20.334 | -4.033471 |
| 2009w39 | 23 | 1 | 1.4 | 10.36185 | 38.64103 | 22.54558 | -1.821889 |
| 2009w40 | 21 | 1 | .5 | 17.45155 | 38.64103 | 29.63529 | 5.267818 |
| 2009w41 | 14 | 1 | .4 | 17.61525 | 38.64103 | 29.79899 | 5.431519 |
| 2009w42 | 22 | 1 | 1.3 | 13.73483 | 38.64103 | 25.91857 | 1.551096 |
| 2009w43 | 6 | 1 | .2 | 16.80921 | 38.64103 | 28.99294 | 4.625471 |
| 2009w44 | 4 | 1 | 1.1 | 9.522714 | 38.64103 | 21.70645 | -2.661021 |
| 2009w45 | 4 | 1 | 6.7 | 5.822924 | 38.64103 | 18.00666 | -6.360811 |
| 2009w46 | 2 | 1 | 53.1 | 5.860552 | 38.64103 | 18.04429 | -6.323183 |
| 2009w47 | 0 | 1 | 3 | 4.26747 | 38.64103 | 16.4512 | -7.916265 |
| 2009w48 | 2 | 1 | 18.4 | 2.50578 | 38.64103 | 14.68951 | -9.677956 |
| 2009w49 | 1 | 1 | .3 | 4.22586 | 38.64103 | 16.40959 | -7.957875 |
| 2009w50 | 9 | 1 | 46.6 | 3.302177 | 38.64103 | 15.48591 | -8.881557 |
| 2009w51 | 1 | 1 | .2 | 7.877776 | 38.64103 | 20.06151 | -4.305959 |
| 2009w52 | 0 | 1 | 93.6 | 4.780506 | 38.64103 | 16.96424 | -7.403229 |
| 2010w1 | 0 | 1 | .3 | 3.300416 | 38.64103 | 15.48415 | -8.883319 |
| 2010w2 | 9 | 1 | 45.9 | 1.504597 | 38.64103 | 13.68833 | -10.67914 |
| 2010w3 | 8 | 1 | 0 | 8.641026 | 38.64103 | 20.82476 | -3.542709 |
| 2010w4 | 5 | 1 | .6 | 7.183892 | 38.64103 | 19.36763 | -4.999843 |
| 2010w5 | 3 | 1 | 13 | 7.460633 | 38.64103 | 19.64437 | -4.723102 |
| 2010w6 | 8 | 1 | 0 | 4.716015 | 38.64103 | 16.89975 | -7.46772 |
| 2010w7 | 4 | 1 | 40.2 | 7.672602 | 38.64103 | 19.85634 | -4.511133 |
| 2010w8 | 3 | 1 | 0 | 6.236386 | 38.64103 | 18.42012 | -5.947349 |
| 2010w9 | 1 | 1 | 0 | 4.281865 | 38.64103 | 16.4656 | -7.90187 |
| 2010w10 | 1 | 1 | 0 | 4.146712 | 38.64103 | 16.33045 | -8.037023 |
| 2010w11 | 1 | 1 | 69.7 | 3.612901 | 38.64103 | 15.79664 | -8.570834 |
| 2010w12 | 12 | 1 | 18.3 | 3.483125 | 38.64103 | 15.66686 | -8.70061 |
| 2010w13 | 6 | 1 | 9.4 | 8.455188 | 38.64103 | 20.63892 | -3.728547 |
| 2010w14 | 9 | 1 | 26.4 | 8.700874 | 38.64103 | 20.88461 | -3.48286 |
| 2010w15 | 2 | 1 | 20.2 | 8.210326 | 38.64103 | 20.39406 | -3.973408 |
| 2010w16 | 1 | 1 | .2 | 4.946771 | 38.64103 | 17.1305 | -7.236964 |
| 2010w17 | 0 | 1 | 34.3 | 3.820419 | 38.64103 | 16.00415 | -8.363316 |
| 2010w18 | 2 | 1 | 165.2 | 4.254548 | 38.64103 | 16.43828 | -7.929187 |
| 2010w19 | 0 | 1 | 16.3 | 3.197691 | 38.64103 | 15.38143 | -8.986044 |
| 2010w20 | 13 | 1 | 20 | 1.525878 | 38.64103 | 13.70961 | -10.65786 |
| 2010w21 | 13 | 1 | 206.2 | 12.99263 | 38.64103 | 25.17637 | .808899 |
| 2010w22 | 6 | 1 | 122.2 | 11.55047 | 38.64103 | 23.73421 | -.6332616 |
| 2010w23 | 6 | 1 | 59.7 | 6.364975 | 38.64103 | 18.54871 | -5.818759 |
| 2010w24 | 16 | 1 | 107.2 | 8.710767 | 38.64103 | 20.8945 | -3.472968 |
| 2010w25 | 14 | 1 | 96.7 | 12.29193 | 38.64103 | 24.47567 | .1081973 |
| 2010w26 | 19 | 1 | 128.1 | 12.78821 | 38.64103 | 24.97195 | .604477 |
| 2010w27 | 2 | 1 | 11.7 | 15.18493 | 38.64103 | 27.36867 | 3.001197 |
| 2010w28 | 0 | 1 | 190.6 | 7.476735 | 38.64103 | 19.66047 | -4.707 |
| 2010w29 | 2 | 1 | 20.6 | 3.906443 | 38.64103 | 16.09018 | -8.277291 |
| 2010w30 | 0 | 1 | .2 | .7003704 | 38.64103 | 12.8841 | -11.48336 |
| 2010w31 | 0 | 1 | 2.1 | 5.179511 | 38.64103 | 17.36325 | -7.004224 |
| 2010w32 | 0 | 1 | 0 | 1.465658 | 38.64103 | 13.64939 | -10.71808 |
| 2010w33 | 11 | 1 | 11.3 | 2.817414 | 38.64103 | 15.00115 | -9.366321 |
| 2010w34 | 1 | 1 | 43.8 | 9.004988 | 38.64103 | 21.18872 | -3.178747 |
| 2010w35 | 0 | 1 | .7 | 4.784708 | 38.64103 | 16.96844 | -7.399027 |
| 2010w36 | 0 | 1 | 15.2 | 2.489064 | 38.64103 | 14.6728 | -9.694671 |
| 2010w37 | 0 | 1 | 1 | 3.033027 | 38.64103 | 15.21676 | -9.150708 |
| 2010w38 | 0 | 1 | 5.4 | 2.148385 | 38.64103 | 14.33212 | -10.03535 |
| 2010w39 | 0 | 1 | 210.9 | 4.689507 | 38.64103 | 16.87324 | -7.494227 |
| 2010w40 | 0 | 1 | 25.3 | 2.541225 | 38.64103 | 14.72496 | -9.642509 |
| 2010w41 | 1 | 1 | 42.9 | .4437172 | 38.64103 | 12.62745 | -11.74002 |
| 2010w42 | 10 | 1 | 40.4 | 5.714435 | 38.64103 | 17.89817 | -6.4693 |
| 2010w43 | 12 | 1 | 26.2 | 6.593256 | 38.64103 | 18.77699 | -5.590478 |
| 2010w44 | 38 | 1 | 28.2 | 11.18612 | 38.64103 | 23.36986 | -.99761 |
| 2010w45 | 13 | 1 | 29.8 | 25.31134 | 38.64103 | 37.49508 | 13.12761 |
| 2010w46 | 21 | 1 | 29.9 | 15.9791 | 38.64103 | 28.16283 | 3.795366 |
| 2010w47 | 2 | 1 | 40.2 | 16.30826 | 38.64103 | 28.49199 | 4.124523 |
| 2010w48 | 3 | 1 | 45.8 | 7.427328 | 38.64103 | 19.61106 | -4.756407 |
| 2010w49 | 0 | 1 | 40.3 | 4.575925 | 38.64103 | 16.75966 | -7.60781 |
| 2010w50 | 1 | 1 | 27.9 | 3.082515 | 38.64103 | 15.26625 | -9.10122 |
| 2010w51 | 0 | 1 | .8 | 2.944306 | 38.64103 | 15.12804 | -9.239429 |
| 2010w52 | 0 | 1 | 57.7 | 3.149833 | 38.64103 | 15.33357 | -9.033902 |
| 2011w1 | 12 | 1 | 0 | 2.67417 | 38.64103 | 14.8579 | -9.509565 |
| 2011w2 | 1 | 1 | 0 | 8.228544 | 38.64103 | 20.41228 | -3.95519 |
| 2011w3 | 0 | 1 | 19.1 | 6.071439 | 38.64103 | 18.25517 | -6.112296 |
| 2011w4 | 0 | 1 | .2 | 2.320385 | 38.64103 | 14.50412 | -9.86335 |
| 2011w5 | 0 | 1 | 0 | 2.443774 | 38.64103 | 14.62751 | -9.739961 |
| 2011w6 | 0 | 1 | 92 | 3.643153 | 38.64103 | 15.82689 | -8.540582 |
| 2011w7 | 4 | 1 | 0 | 2.388431 | 38.64103 | 14.57217 | -9.795303 |
| 2011w8 | 1 | 1 | 10.9 | 3.741212 | 38.64103 | 15.92495 | -8.442523 |
| 2011w9 | 0 | 1 | .5 | 4.935985 | 38.64103 | 17.11972 | -7.24775 |
| 2011w10 | 0 | 1 | 0 | 1.936068 | 38.64103 | 14.1198 | -10.24767 |
| 2011w11 | 0 | 1 | 4.8 | 2.910864 | 38.64103 | 15.0946 | -9.272871 |
| 2011w12 | 0 | 1 | 37.1 | 2.857552 | 38.64103 | 15.04129 | -9.326182 |
| 2011w13 | 0 | 1 | 10.2 | 2.596863 | 38.64103 | 14.7806 | -9.586872 |
| 2011w14 | 0 | 1 | 31.9 | 2.467796 | 38.64103 | 14.65153 | -9.71594 |
| 2011w15 | 0 | 1 | 11.9 | 2.967502 | 38.64103 | 15.15124 | -9.216232 |
| 2011w16 | 0 | 1 | 5.1 | 2.091243 | 38.64103 | 14.27498 | -10.09249 |
| 2011w17 | 0 | 1 | 24.9 | 3.037316 | 38.64103 | 15.22105 | -9.146419 |
| 2011w18 | 1 | 1 | 43.3 | 2.827467 | 38.64103 | 15.0112 | -9.356267 |
| 2011w19 | 0 | 1 | 118.1 | 3.864143 | 38.64103 | 16.04788 | -8.319592 |
| 2011w20 | 0 | 1 | 63.1 | 3.013118 | 38.64103 | 15.19685 | -9.170616 |
| 2011w21 | 2 | 1 | 68.1 | 2.061881 | 38.64103 | 14.24562 | -10.12185 |
| 2011w22 | 1 | 1 | 194.8 | 5.752141 | 38.64103 | 17.93587 | -6.431594 |
| 2011w23 | 14 | 1 | 86.1 | 3.26965 | 38.64103 | 15.45338 | -8.914085 |
| 2011w24 | 17 | 1 | 43.9 | 8.843329 | 38.64103 | 21.02706 | -3.340405 |
| 2011w25 | 17 | 1 | 89.4 | 15.85752 | 38.64103 | 28.04125 | 3.673785 |
| 2011w26 | 3 | 1 | 93 | 14.59545 | 38.64103 | 26.77919 | 2.411718 |
| 2011w27 | 15 | 1 | 1.8 | 6.18584 | 38.64103 | 18.36958 | -5.997895 |
| 2011w28 | 19 | 1 | 63.7 | 11.56876 | 38.64103 | 23.75249 | -.6149787 |
| 2011w29 | 21 | 1 | 15.8 | 16.00219 | 38.64103 | 28.18592 | 3.818453 |
| 2011w30 | 16 | 1 | 8.1 | 15.88457 | 38.64103 | 28.0683 | 3.700835 |
| 2011w31 | 11 | 1 | 3.5 | 15.53559 | 38.64103 | 27.71933 | 3.351859 |
| 2011w32 | 8 | 1 | 1.9 | 10.86896 | 38.64103 | 23.05269 | -1.314776 |
| 2011w33 | 14 | 1 | 4.5 | 8.828151 | 38.64103 | 21.01188 | -3.355584 |
| 2011w34 | 20 | 1 | 12.2 | 11.59282 | 38.64103 | 23.77656 | -.5909127 |
| 2011w35 | 23 | 1 | 11 | 15.77447 | 38.64103 | 27.95821 | 3.590739 |
| 2011w36 | 28 | 1 | 4.8 | 18.29674 | 38.64103 | 30.48047 | 6.113003 |
| 2011w37 | 47 | 1 | 5.7 | 21.59864 | 38.64103 | 33.78237 | 9.414902 |
| 2011w38 | 45 | 1 | 19.8 | 32.82429 | 38.64103 | 45.00803 | 20.64056 |
| 2011w39 | 57 | 1 | 54.7 | 35.18413 | 38.64103 | 47.36786 | 23.00039 |
| 2011w40 | 18 | 1 | 23.1 | 40.90716 | 38.64103 | 53.0909 | 28.72343 |
| 2011w41 | 18 | 1 | 145 | 22.71081 | 38.64103 | 34.89454 | 10.52707 |
| 2011w42 | 52 | 1 | 25.1 | 15.72865 | 38.64103 | 27.91239 | 3.544919 |
| 2011w43 | 24 | 1 | 41.9 | 32.16546 | 38.64103 | 44.34919 | 19.98172 |
| 2011w44 | 9 | 1 | 14.7 | 25.81207 | 38.64103 | 37.9958 | 13.62834 |
| 2011w45 | 12 | 1 | 32.3 | 10.53775 | 38.64103 | 22.72148 | -1.645989 |
| 2011w46 | 10 | 1 | 49 | 11.38646 | 38.64103 | 23.5702 | -.7972716 |
| 2011w47 | 17 | 1 | 19.6 | 9.701152 | 38.64103 | 21.88489 | -2.482583 |
| 2011w48 | 19 | 1 | 25.7 | 13.40322 | 38.64103 | 25.58695 | 1.219484 |
| 2011w49 | 29 | 1 | 33 | 16.13472 | 38.64103 | 28.31845 | 3.950985 |
| 2011w50 | 24 | 1 | 0 | 21.03132 | 38.64103 | 33.21506 | 8.847589 |
| 2011w51 | 20 | 1 | 37.6 | 20.60851 | 38.64103 | 32.79224 | 8.42477 |
| 2011w52 | 6 | 1 | 2 | 17.67497 | 38.64103 | 29.85871 | 5.49124 |
| 2012w1 | 0 | 1 | 0 | 8.527857 | 38.64103 | 20.71159 | -3.655878 |
| 2012w2 | 7 | 1 | 0 | 4.094124 | 38.64103 | 16.27786 | -8.08961 |
| 2012w3 | 6 | 1 | 5.1 | 6.156423 | 38.64103 | 18.34016 | -6.027312 |
| 2012w4 | 8 | 1 | 1.3 | 7.053487 | 38.64103 | 19.23722 | -5.130248 |
| 2012w5 | 3 | 1 | 0 | 7.860315 | 38.64103 | 20.04405 | -4.32342 |
| 2012w6 | 0 | 1 | 18 | 5.751192 | 38.64103 | 17.93493 | -6.432543 |
| 2012w7 | 20 | 1 | 0 | 3.057668 | 38.64103 | 15.2414 | -9.126067 |
| 2012w8 | 9 | 1 | 3.1 | 13.17852 | 38.64103 | 25.36226 | .9947902 |
| 2012w9 | 3 | 1 | 24.9 | 11.25352 | 38.64103 | 23.43725 | -.9302167 |
| 2012w10 | 11 | 1 | 35.1 | 5.844263 | 38.64103 | 18.028 | -6.339472 |
| 2012w11 | 12 | 1 | 32.1 | 9.051753 | 38.64103 | 21.23549 | -3.131982 |
| 2012w12 | 9 | 1 | 4.1 | 10.78939 | 38.64103 | 22.97312 | -1.394347 |
| 2012w13 | 1 | 1 | 0 | 9.317709 | 38.64103 | 21.50144 | -2.866026 |
| 2012w14 | 1 | 1 | 2 | 4.787368 | 38.64103 | 16.9711 | -7.396367 |
| 2012w15 | 0 | 1 | 18.6 | 3.333817 | 38.64103 | 15.51755 | -8.849917 |
| 2012w16 | 7 | 1 | 39.1 | 3.092651 | 38.64103 | 15.27639 | -9.091084 |
| 2012w17 | 0 | 1 | 10.9 | 6.242335 | 38.64103 | 18.42607 | -5.9414 |
| 2012w18 | 2 | 1 | 43.3 | 3.87179 | 38.64103 | 16.05552 | -8.311945 |
| 2012w19 | 5 | 1 | 95 | 4.692359 | 38.64103 | 16.87609 | -7.491376 |
| 2012w20 | 3 | 1 | 75.2 | 5.579309 | 38.64103 | 17.76304 | -6.604426 |
| 2012w21 | 7 | 1 | 101.1 | 5.258423 | 38.64103 | 17.44216 | -6.925312 |
| 2012w22 | 6 | 1 | 66.9 | 7.359799 | 38.64103 | 19.54353 | -4.823936 |
| 2012w23 | 3 | 1 | 38.1 | 6.360008 | 38.64103 | 18.54374 | -5.823727 |
| 2012w24 | 3 | 1 | 117.6 | 6.275021 | 38.64103 | 18.45876 | -5.908714 |
| 2012w25 | 5 | 1 | 118.9 | 5.453895 | 38.64103 | 17.63763 | -6.72984 |
| 2012w26 | 3 | 1 | 86.1 | 5.197494 | 38.64103 | 17.38123 | -6.986241 |
| 2012w27 | 3 | 1 | 134.9 | 6.032186 | 38.64103 | 18.21592 | -6.151549 |
| 2012w28 | 9 | 1 | 6.1 | 4.302137 | 38.64103 | 16.48587 | -7.881598 |
| 2012w29 | 4 | 1 | 15.8 | 6.812698 | 38.64103 | 18.99643 | -5.371037 |
| 2012w30 | 9 | 1 | 5.3 | 7.478147 | 38.64103 | 19.66188 | -4.705588 |
| 2012w31 | 5 | 1 | 1.5 | 7.169458 | 38.64103 | 19.35319 | -5.014277 |
| 2012w32 | 0 | 1 | 0 | 7.117085 | 38.64103 | 19.30082 | -5.06665 |
| 2012w33 | 3 | 1 | 0 | 3.389243 | 38.64103 | 15.57298 | -8.794492 |
| 2012w34 | 3 | 1 | 6.6 | 4.248536 | 38.64103 | 16.43227 | -7.935199 |
| 2012w35 | 1 | 1 | 11 | 4.802164 | 38.64103 | 16.9859 | -7.381571 |
| 2012w36 | 0 | 1 | 4.8 | 3.585854 | 38.64103 | 15.76959 | -8.59788 |
| 2012w37 | 1 | 1 | 5.7 | 2.745916 | 38.64103 | 14.92965 | -9.437819 |
| 2012w38 | 1 | 1 | 19.8 | 3.322465 | 38.64103 | 15.5062 | -8.86127 |
| 2012w39 | 0 | 1 | 54.7 | 3.683151 | 38.64103 | 15.86689 | -8.500584 |
| 2012w40 | 1 | 1 | 23.1 | 2.73989 | 38.64103 | 14.92362 | -9.443845 |
| 2012w41 | 5 | 1 | 24.6 | 2.86984 | 38.64103 | 15.05358 | -9.313894 |
| 2012w42 | 7 | 1 | 25.1 | 5.912848 | 38.64103 | 18.09658 | -6.270887 |
| 2012w43 | 7 | 1 | 28.7 | 7.056806 | 38.64103 | 19.24054 | -5.126929 |
| 2012w44 | 5 | 1 | 30.6 | 7.717543 | 38.64103 | 19.90128 | -4.466192 |
| 2012w45 | 7 | 1 | 20.3 | 6.450764 | 38.64103 | 18.6345 | -5.732971 |
| 2012w46 | 10 | 1 | 53.9 | 7.501375 | 38.64103 | 19.68511 | -4.68236 |
| 2012w47 | 2 | 1 | 38.1 | 9.428728 | 38.64103 | 21.61246 | -2.755007 |
| 2012w48 | 6 | 1 | 25.7 | 4.970772 | 38.64103 | 17.15451 | -7.212963 |
| 2012w49 | 2 | 1 | 5.1 | 6.293041 | 38.64103 | 18.47678 | -5.890694 |
| 2012w50 | 2 | 1 | 55.1 | 4.952806 | 38.64103 | 17.13654 | -7.230929 |
| 2012w51 | 3 | 1 | 9.6 | 4.121331 | 38.64103 | 16.30507 | -8.062404 |
| 2012w52 | 2 | 1 | 38.1 | 4.144044 | 38.64103 | 16.32778 | -8.03969 |
